# Effects of a novel medium–long–medium‐type structured lipid synthesized using a two‐step enzymatic method on lipid metabolism and obesity protection in experimental mice

**DOI:** 10.1002/fsn3.3410

**Published:** 2023-05-11

**Authors:** Yahan Zhou, Yuejie Xie, Zhongming Wang, Cao Wang, Qiang Wang

**Affiliations:** ^1^ Beijing Advanced Innovation Center for Food Nutrition and Human Health Beijing Technology and Business University Beijing China; ^2^ Key Laboratory of Lipid Resources Utilization and Children's Daily Chemicals Chongqing University of Education Chongqing China

**Keywords:** energy supply, enzymatic hydrolysis, fatty acids, lipid metabolism, obesity

## Abstract

We synthesized a novel, structured lipid containing caprylic acid at its *sn*‐1,3 position and docosahexaenoic acid at its *sn*‐2 position (1,3C‐2D‐TAG) by using a two‐step enzymatic method and then investigated the relationship between the types of fatty acids in the structured lipid and their physiological functions. Furthermore, we compared the effects of similarly structured lipids on postprandial lipid metabolism and obesity protection. The results revealed that the novel structured lipid synthesized using the enzymatic method not only exhibited better physical properties than traditional oils but also had high oxidation stability and crystallization degree. In terms of physiological function, the low‐dose 1,3C‐2D‐TAG group exhibited higher cholesterol and triglyceride levels, lower kidney weight or body weight, and higher serum aspartate aminotransferase and blood urea nitrogen levels than control group, whereas the high‐dose 1,3C‐2D‐TAG group exhibited higher triglyceride levels. Moreover, the medium‐dose 1,3C‐2D‐TAG group had remarkably higher high‐density lipoprotein cholesterol levels and lower low‐density lipoprotein cholesterol levels than the high‐lipid, control, and 1,2,3C‐TAG groups; however, the cholesterol and triglyceride levels and kidney and liver functions did not differ significantly among these groups. The study results suggest that 1,3C‐2D‐TAG can not only facilitate the effective utilization of the energy supplied by medium‐chain fatty acids but also help overcome difficulties in the absorption of long‐chain polyunsaturated fatty acids.

## Graphical abstract

- A new structured lipid with DHA onto *sn*‐2 position was synthesized by a two‐step enzymatic method.
- The new‐style structured lipid synthesized by enzymatic method had high oxidation stability and crystallization degree.
- New‐style structured lipids significantly reduce diet‐induced leptin concentration and alleviate obesity‐induced leptin resistance.
- Prepared structured lipid can supply energy timely than conventional lipid.

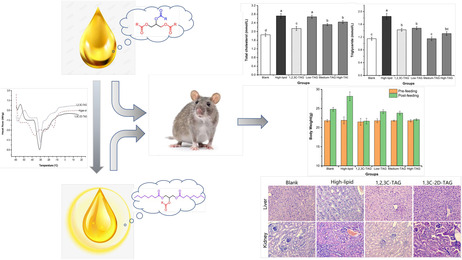

## 1 INTRODUCTION

Obesity can significantly increase the risks of diabetes, atherosclerosis, hyperlipidemia, hypertension, and other diseases; therefore, it has emerged as a major public health concern worldwide (Moreira et al., 2017). Obesity is mainly caused by the excessive intake of dietary energy but insufficient calorie consumption, resulting in systematic accumulation of fat in the body (Kok et al., 2018). Obesity can be effectively prevented and controlled by optimizing the type of fats in the diet and developing low‐calorie fats. The directional modification technology of oil not only modifies specific fatty acids in natural oils and fats, thereby allowing them to fully exert their corresponding nutritional characteristics but also helps to prevent certain diseases or improve metabolic conditions. Therefore, it has received considerable attention from researchers in the fields of oil technology and nutrition (Kim & Akoh, 2015; Korma et al., 2018).

Structured lipids are a class of triglycerides with specific molecular structures and functions that are generated by chemically or enzymatically modifying fatty acids or different positions and distributions on the carbon chain skeleton of triglycerides (Costa et al., 2018; More, Gogate, et al., 2018; Zhang et al., 2018). The differences in structured lipids lie not only in the types of fatty acids attached to the triglyceride (TAG) skeleton but also in the random or selective localization of fatty acids on the skeleton (e.g., lateral *sn‐1* and *sn‐3* or middle *sn‐2*). Concatenating natural lipids with fatty acids with special nutritional or physiological functions at a specific location of the TAG skeleton results in the generation of structured lipids with optimal composition and structure; these lipids can not only maintain a part or all the properties of natural oil but also maximize fatty acid functions (Bastos et al., 2009; Moreira et al., 2017).

Researchers have attempted to connect the omega‐3 and omega‐6 series of free unsaturated fatty acids to the specific position of the glycerol carbon chain skeleton to increase the stability and improve the digestibility and absorption rate of fatty acids in vivo (Morre et al., 2010; Senanayake & Shahidi, 2002; Sheppard & Cheatham, 2018). *n*‐3 polyunsaturated fatty acids (PUFA) have been widely used as a functional component in liquid and solid fortified foods such as infant formula (Pusceddu et al., 2015; Rimm et al., 2018). One such PUFA is docosahexaenoic acid (DHA), which exhibits neuroprotective and cardioprotective effects against several diseases, including Alzheimer's disease and cardiovascular diseases (Endo & Arita, 2016; Pan et al., 2015). Furthermore, the human body can better absorb lipids with PUFA at *sn*‐2 position than 2‐monoacylglycerols (2‐MAG). A review has shown that DHA/eicosapentaenoic acid (EPA)‐enriched glycerophospholipids are beneficial for human health as they improve brain function and exert an anticancer effect (Zhang et al., 2019).

Although several studies have assessed the absorption rate and physiological functions of structured lipids, they have mainly focused on the effects of *sn*‐2 monoglycerides on improving their digestibility and absorption rate. The effect of *sn*‐2 long‐chain unsaturated fatty acid triglycerides on blood lipid metabolism in vivo remains unclear. Some studies have compared the intestinal absorption rate of different fatty acid compositions and TAG structures in rats and revealed that the absorption rate of *sn*‐1,3 saturated fatty acids in the intestinal tract after a meal is considerably lower than that of unsaturated fatty acids at *sn*‐2 position (Porsgaard & Høy, 2000). When medium‐chain fatty acids are grafted at *sn*‐1,3 position of triglycerides, the triglycerides can be quickly absorbed and converted into free fatty acids via the portal vein; on the other hand, inserting long‐chain PUFAs at *sn*‐2 position can significantly improve the oxidation stability and absorption rate of the generated 2‐MAG (Kahveci et al., 2015). Compared with long‐chain fatty acids, medium‐chain fatty acids can more quickly supply energy to the body and are not mixed with chyle. Therefore, most medium‐chain fatty acids are not stored in the adipose tissue; at an appropriate amount, they can reduce the body's demand for glucose. In general, the fatty acids obtained after triglyceride digestion can only be absorbed in the form of nonesterified free fatty acids or 2‐MAG. When nonabsorbable fatty acids are esterified at *sn*‐2 position, the generated 2‐MAG can be directly used by intestinal cells to synthesize triglycerides that are involved in the assembly of chyle microparticles, with considerable improvements in its absorption rate (Armand, 2008). Moreover, incorporating *n‐3* fatty acids with higher bioactivities at *sn*‐2 position of triglycerides can promote the health benefits of functional fatty acids. Therefore, incorporating essential fatty acids and medium‐chain structural lipids into triglycerides allows the molecules to exert dual effect: nutrition and energy intake control. Therefore, it is important to fully understand the relationship between the physiological functions and structural characteristics of structured lipids.

Structured lipids offer many remarkable advantages, including improving immunity, preventing obesity and cancer, and alleviating nutritional disorders; all of these are closely related to the structurally linked functional fatty acids. The improved design of essential fatty acids and medium‐chain structural esters in triglycerides can allow them to satisfy the dual purpose (nutrition and energy intake control) and thus contribute to the complete understanding of the relationship among the physicochemical properties, physiological effects, and structural characteristics of structured lipids.

In the present study, to investigate the effects of structured lipids on lipid metabolism and obesity protection, we fabricated a novel structured lipid (medium–long–medium [MLM]‐type) mainly containing caprylic acid (medium‐chain fatty acid; C8:0) at *sn*‐1,3 position and DHA (long‐chain fatty acid) at *sn*‐2 position; the lipid was named 1,3C‐2D‐TAG. We studied the effects of this novel structured lipid on the prevention of postprandial lipid metabolism and obesity in mice. Our study will help clarify the intrinsic relationship between the types of fatty acids in structured lipids and their physiological functions.

## 2 MATERIALS AND METHODS

### 2.1 Materials

A mixed fatty acid methyl ester was purchased from Sigma‐Aldrich (Sigma, St Louis, MO, USA). Immobilized lipase from *Rhizomucor miehei* (Lipozyme RM IM) was purchased from Novozymes A/S (Bagsvaerd, Denmark). Dry ethanol, n‐hexane, acetone, chloroform, absolute ethanol, NaCl, and acetonitrile of high‐performance liquid chromatography (HPLC) or analytical grade were obtained from Thermo Fisher Scientific (Waltham, MA, USA).

### 2.2 Preparation of MLM‐type structured lipids using the two‐step enzymatic hydrolysis method

Briefly, 0.9 g of oil and 3 g of anhydrous ethanol were weighed and added to a 50‐mL beaker. Then, 0.4 g of Lipozyme RM IM was added, and the mixture was stirred at 200 rpm using a magnetic stirrer (IKA, Staufen, Germany) for 5 h at 40°C. After removing lipase, the lower alcohol solution was further extracted with 15 mL of n‐hexane by oscillating violently for 2 min and then leaving it undisturbed. After the formation of two layers, the ethanol phase containing 2‐MAGs was collected. MAG (100 g) was mixed with 150 g of octanoic acid, 25 g of lipase, 1000 mL of n‐hexane, and 2 g of a 4A molecular sieve. The mixture was magnetically stirred at 200 rpm for 5 h at 40°C. After centrifuging the samples at 500 rpm for 10 min, lipase was separated from the sample to obtain the crude MLM sample. This lipid sample was further purified on a chromatography column and then eluted with n‐hexane/diethyl ether (95/5, v/v) solution. The eluted fractions were analyzed using thin‐layer chromatography (TLC) and HPLC. The amounts of ingredients used in the animal feed were 100 times higher than those required, and the feed was stored at −20°C.

### 2.3 Analysis of the structured lipid using HPLC

The MLM‐type structured lipid product was subjected to HPLC analysis, as described by More, Waghmare, et al. (2018). Briefly, 2 μL of the sample was injected into an HPLC column equipped with a Ultraviolet detector (254 nm) (Shimadzu, Kyoto, Japan). The flow rate was maintained at 1 mL/min, and the mobile phase was acetonitrile/acetic acid (9/1, v/v). The composition of the mixture was determined based on retention time and peak order, and the corresponding relative contents were calculated from peak areas.

### 2.4 Determination of the composition of *sn*‐2 fatty acids using gas chromatography–flame ionization detection (GC–FID)

First, 2 mL of 0.5 M NaOH–CH_3_OH was saponified with 3 g of the samples at 60°C for 30 min and then allowed to react with 14% boron trifluoride at 60°C for 5 min. After completion of the reaction, methyl fatty acid ester was extracted using approximately 2 mL of hexane, followed by calculating the molar percentage of fatty acid composition at the *sn*‐2 position of MAG.

The GC conditions were as follows: chromatographic column, FFAP capillary column (30 m × 0.25 mm × 0.5 μm; Agilent, Santa Clara, CA, USA); detector, FID; carrier gas, N_2_; flow rate, 1.0 mL/min; inlet pressure, 25 psi; and shunt ratio, 30:1. The initial furnace temperature was set at 140°C for 1 min and then increased to 230°C at a rate of 10°C/min and held for 8 min. The detector temperature was maintained at 280°C. Peak time and relative peak area were used for the quantitative and qualitative analyses of fatty acid methyl esters, respectively.

### 2.5 Animals and treatment

Briefly, 60 six‐week‐old C57BL/6 mice (30 males and 30 females; weight = 25 ± 2 g), provided by the Experimental Animal Center of Army Characteristic Medical Center (Certificate No. SCXK (Jing) 2019–0010, Beijing, China), were used. All animals were housed in microisolator cages in a light‐controlled room under a 12/12‐h light/dark cycle at a controlled temperature of 25°C and relative humidity of 50%. The animals were given ad libitum access to food and water according to the Guide for the Care and Use of Laboratory Animals. Caution was exercised to avoid unnecessary pain to the animals.

The animals were divided into six groups, with each group having 10 mice. The groups were as follows: the control, high‐lipid, 1,2,3C‐TAG, and 1,3C‐2D‐TAG groups; the 1,3C‐2D‐TAG group was further divided into the 1,3C‐2D‐TAG low‐dose (20 g/kg/day), medium‐dose (50 g/kg/day), and high‐dose (100 g/kg/day) groups. The compositions of the feeds fed to different groups are shown in Table 1. After 5 days of adaptive feeding, the mice in the different groups were given ad libitum access to the feeds (listed in Table 1) and water for 6 days. Food intake was measured, and the feed and water were refilled every day. The weights of the mice were measured once a week. After 6 days of feeding, the mice were starved for 24 h, followed by weight measurements. Subsequently, the animals were anesthetized by intraperitoneally injecting 3% chloral hydrate and then sacrificed. Blood samples, liver, kidneys, testis, kidney organs, and fat tissues were collected. The ratios of liver weight (LW)/body weight (BW) and kidney weight/BW were calculated.

**TABLE 1:** Composition of feeds fed to different groups of mice (wt %).

Group	Control group	High lipid group	1,2,3C‐TAG group	1,3C‐2D‐TAG group		
				Low‐dose group	Moderate‐dose group	High‐dose group
Basal feed	88.5	88.5	88.5	88.5	88.5	88.5
Lard		10				
Cholesterol	1	1	1	1	1	1
Pig bile salts	0.5	0.5	0.5	0.5	0.5	0.5
Soybean oil	10			8	5	
Caprylic triglyceride			10			
1,3C‐2D‐TAG				2	5	10

### 2.6 Measurement of serum factors

The serum levels of total cholesterol, triglycerides, high‐density lipoprotein cholesterol (HDL‐C), low‐density lipoprotein cholesterol (LDL‐C), alanine aminotransferase (ALT), aspartate aminotransferase (AST), and blood urea nitrogen (BUN) were measured using the BC‐240‐VET animal automatic biochemical analyzer (Shenzhen Mindray Biomedical Electronic Technology Co. Ltd., Shenzhen, China).

### 2.7 Histological analysis

Histological analysis was conducted using hematoxylin and eosin (H&E) staining. The collected tissues were stored at −20°C before use. Briefly, all tissues were fixed with 10% formalin, embedded with paraffin, and then sectioned. Thereafter, the sections were stained with H&E and observed under a light microscope (Zeiss, Heidelberg, Germany).

### 2.8 Statistical analysis

All experiments were conducted in triplicate. The measured data are expressed as the mean ± SD. Data were analyzed via variance analysis, and multiple comparisons were conducted using the least significant difference method. Differences were considered statistically significant when *p* value was <.05. All calculations were performed using Origin 8.5.

## 3 RESULTS AND DISCUSSION

### 3.1 TLC analysis of the MLM‐type structured lipid

**FIGURE 1:**
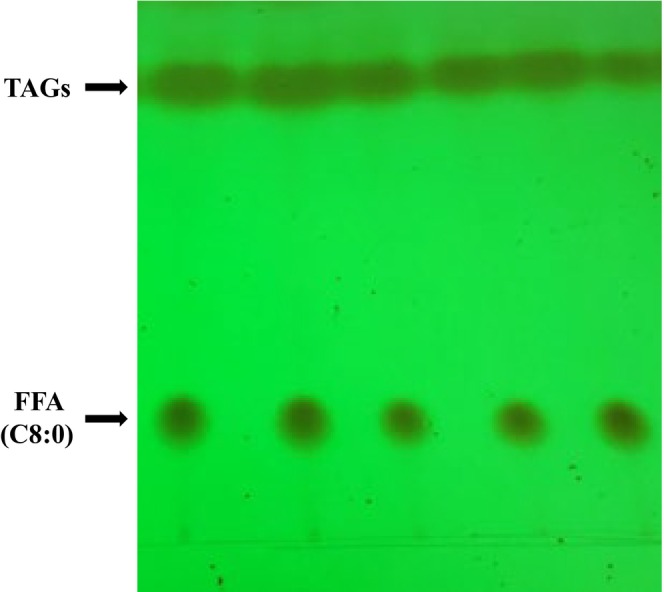
Thin‐layer chromatography of 1,3C‐2D‐TAG structured lipids.

When synthesizing 1,3C‐2D‐TAG in anhydrous ethanol, C8:0 was used as the source of medium‐chain fatty acids at *sn*‐1,3 position of 2D‐MAG‐structured glycerin. MLM‐type *sn*‐2 long‐chain PUFA triglycerides were synthesized using a two‐step enzymatic method. During synthesis of the structured lipid, in addition to the target triglycerides, some by‐products such as monoglycerides, diglycerides, and free fatty acids were also generated. These products were identified on the TLC plate as strips from the bottom to the top of the plate according to their corresponding molecular weights. The spot indicating triglyceride in the TLC of 1,3C‐2D‐TAG was observed at a retention factor (Rf) of 0.83 (Figure 1), which is within the range of triglyceride‐specific shift values (Rf = 0.70–0.85). This finding preliminarily confirmed that the synthesis route and method used are effective. In addition, the spot in the TLC plate at an Rf of 0.23 was identified as octanoic acid, which was not fully involved in the reaction by GC. Some studies have reported that 37.80% of ABA triglycerides can be obtained via enzymatic modification with EPA and trioctyl triglycerides (Pina‐Rodriguez & Akoh, 2010). Studies have also reported that the ethyl ester mixture of EPA can more effectively be combined into structured lipids than its respective fatty acids (Pina‐Rodriguez & Akoh, 2010). Xu (2000) used lipase (Lipozyme RM IM) to catalyze the reaction between C8:0 and mustard oil and obtained products containing triglycerides with octanoic acid at their *sn*‐1,3 position with a mass fraction of 40.10%.

### 3.2 Composition and content of fatty acids in the structured lipid

Table 2 presents the composition and content of natural and lipase‐modified fatty acids in solvent‐free media. The main fatty acids in algal oil were C22:6 (44.37%) and C16:0 (27.50%); other fatty acids such as C14:0, C18:1, and C22:5 were also present with low contents of 6.21%, 5.37%, and 7.42%, respectively. PUFA content at *sn*‐1,3 position (54.26%) was lower than that at *sn*‐2 position (64.29%). In particular, 45.87% of DHA was mainly concentrated at *sn*‐1,3 position of natural algal oil. In addition, the distribution of monounsaturated fatty acids at *sn*‐1,3 position, which was only 1.65%, was considerably lower than that of PUFAs (52.25%), indicating that natural algal oil is one of the ideal materials for producing MLM‐type structured lipids with PUFAs.

**TABLE 2:** Composition and position of fatty acid in algae oil (%).

Fatty acid	Unmodified oil			Modified oil		
	Total fatty acid	*sn*‐2 position	*sn*‐1,3 position	Total fatty acid	*sn*‐2 position	*sn*‐1,3 position
C8:0	NF	NF	NF	20.35 ± 1.53^a^	2.39 ± 0.08^a^	29.33 ± 1.79^a^
C14:0	6.21 ± 0.22^a^	3.85 ± 0.17^a^	6.89 ± 0.21^a^	3.64 ± 0.21^b^	5.61 ± 0.46^b^	2.66 ± 0.12^b^
C16:0	27.50 ± 0.94^a^	18.85 ± 1.32^a^	32.83 ± 2.03^a^	24.39 ± 2.48^a^	28.83 ± 3.27^b^	22.17 ± 3.26^b^
C16:1	1.73 ± 0.15^a^	1.47 ± 0.11^a^	1.36 ± 0.09^a^	1.15 ± 0.11^b^	1.60 ± 0.15^a^	0.93 ± 0.06^b^
C18:0	2.99 ± 0.22^a^	5.46 ± 0.08^a^	3.26 ± 0.25^a^	2.36 ± 0.30^a^	5.07 ± 0.36^a^	1.01 ± 0.03^b^
C18:1	5.37 ± 0.01^a^	3.54 ± 0.26^a^	0.29 ± 0.07^a^	1.63 ± 0.09^b^	2.25 ± 0.19^b^	1.32 ± 0.10^b^
C18:2 n‐6	0.51 ± 0.02^a^	0.63 ± 0.12^a^	0.45 ± 0.06^a^	0.40 ± 0.03^b^	0.99 ± 0.10^b^	0.11 ± 0.04^b^
C18:3 n‐3	0.46 ± 0.01^a^	NF	0.69 ± 0.04^a^	0.35 ± 0.02^b^	NF	0.53 ± 0.02^b^
C20:4 n‐3	0.24 ± 0.02^a^	0.36 ± 0.02^a^	0.18 ± 0.02^a^	0.42 ± 0.10^b^	1.01 ± 0.07^b^	0.13 ± 0.01^b^
C20:5 n‐3	1.26 ± 0.31^a^	4.58 ± 0.01^a^	2.60 ± 0.25^a^	2.72 ± 0.08^b^	5.01 ± 0.33^a^	1.58 ± 0.16^b^
C22:5 n‐6	7.42 ± 0.12^a^	11.34 ± 0.15^a^	2.46 ± 0.18^a^	2.15 ± 0.34^b^	4.38 ± 0.24^b^	1.04 ± 0.11^b^
C22:6 n‐3	44.37 ± 2.75^a^	47.38 ± 4.69^a^	45.87 ± 3.95^a^	38.14 ± 3.55^b^	39.38 ± 4.63^b^	37.52 ± 4.52^b^
∑SFA	36.71 ± 3.94^a^	28.16 ± 1.20^a^	42.97 ± 2.36^a^	50.74 ± 4.64^b^	41.90 ± 4.99^b^	55.16 ± 6.14^b^
∑MUFA	7.13 ± 0.20^a^	5.01 ± 0.53^a^	1.65 ± 0.10^a^	2.78 ± 1.81^b^	3.85 ± 0.24^b^	2.25 ± 0.23^b^
∑PUFA	54.26 ± 3.34^a^	64.29 ± 4.85^a^	52.25 ± 3.75^a^	44.18 ± 5.12^b^	50.77 ± 4.96^b^	40.89 ± 4.86^b^
∑n‐3 PUFA	46.33 ± 4.01^a^	52.32 ± 5.17^a^	43.26 ± 3.68^a^	41.63 ± 3.44^a^	45.40 ± 3.42^b^	39.76 ± 4.38^b^

*Note*: Values are means ± standard deviation. Mean values in the same row with different superscript letters are significantly different (*p* < .05).

Abbreviation: NF, Not found.

As shown in Table 2, the modified structural esters mainly included C22:6, C16:0, and C8:0, with contents of 38.14%, 24.39%, and 20.35%, respectively. The content of octanoic acid was significantly higher (*p* < .05) in the structural ester than in natural algal oil. The content of the modified structural esters of *sn*‐1,3 DHA decreased from 45.87% to 37.52% (*p* < .05), whereas that of *sn*‐1,3 DHA increased to 29.33%. However, the content of DHA at *sn*‐2 position did not change significantly (39.38%) and that of octanoic acid at the same position was only 2.39%. Abed et al. (2018) reported that after lipase catalysis, the content of long‐chain ARA at *sn*‐2 position significantly increased from 44.53% to 49.45%. After the addition of octanoic acid, the content of structural esters at *sn*‐1 position of lignin acid and palmitic acid decreased from 16.30% to 8.65% and 11.60% to 4.09%, respectively. This suggests that the MLM structural esters of *sn*‐2 long‐chain PUFAs, which have better structural properties, can be obtained from octanoic acid and algal oil, with Lipozyme RM IM as the catalyst.

### 3.3 Physicochemical properties of the modified structured lipid

The physicochemical properties of oils (algal oil and the structured lipid) before and after modification are presented in Table 3. The refractive index and specific gravity of the oils before and after modification did not differ significantly (*p* > .05). Algal oil had a significantly higher iodine value than the ester‐modified oil (*p* < .05), possibly because the content of the modified samples with long‐chain unsaturated fatty acids had decreased and the composition and content of total fatty acids were altered. This also suggests that the structure containing a higher algal oil content has a higher oxidation stability (Sivakanthan et al., 2019). Rocha‐Uribe and Hernandez (2008) elucidated the physicochemical properties of structural esters synthesized from conjugated linoleic acid and coconut oil and suggested that the oxidation stability of structural esters is related to the content of conjugated linoleic acid and location of fatty acids, both of which affect the physical properties of structural fats. Furthermore, Hamam and Shahidi (2005) studied the enzymatic modification of decanoic acid and a commercial omega‐3 oil derived from the merozoite of microalgae. The obtained structural esters were found to contain decanoic acid at *sn*‐1,3 position, and DHA was found to be esterified at *sn*‐2 position.

**TABLE 3:** Physicochemical properties of algal oil and structured lipids.

Physicochemical properties	Algal oil	Structured lipids
Refractive index (25°C)	1.56 ± 0.01^a^	1.39 ± 0.02^a^
Specific gravity (25°C, g/Ml)	0.95 ± 0.07^a^	0.92 ± 0.01^a^
Saponification value (mg KOH/g)	180.06 ± 9.66^a^	249.52 ± 4.31^b^
Iodine value (g I_2_/100 g)	151.39 ± 6.79^a^	70.69 ± 1.65^b^
Calorific value (kJ/g)	42.62 ± 2.38^a^	33.23 ± 3.44^b^

*Note*: Values are means ± standard deviation. Mean values in the same row with different superscript letters are significantly different (*p* < .05).

Calorific value is an important index that reflects the amount of energy supplied by fats and oils to the body. The intake of certain amounts of fats with a low calorific value can considerably control the prevalence of obesity and cardiovascular and cerebrovascular diseases (Berry, 2009). Table 3 demonstrates that the calorific value of the modified algal oil was reduced by 22.41% (*p* < .05). A possible reason for this is that the *sn*‐1,3 position is easily accessible to the saturated fatty acid chain, resulting in a decrease in the conversion rate of thermal energy of the oil. Norizzah et al. (2015) reported that structured lipids have lower calorific values than a mixture of enzyme‐catalyzed esterified palmitostearic acid and oleic acid. Liu et al. (2013) synthesized SLS‐type lipids (purity = 94.3%) through secondary enzymatic hydrolysis and found that the calorific value (21.12 kJ/g) of the lipids was only 55% of that of the original soybean oil. These results suggest that the ester not only retains the nutritional and physical properties of traditional oils but also exerts high oxidation stability, possibly replacing traditional oils.

### 3.4 DSC analysis of the MLM‐type structured lipids before and after modification

**FIGURE 2:**
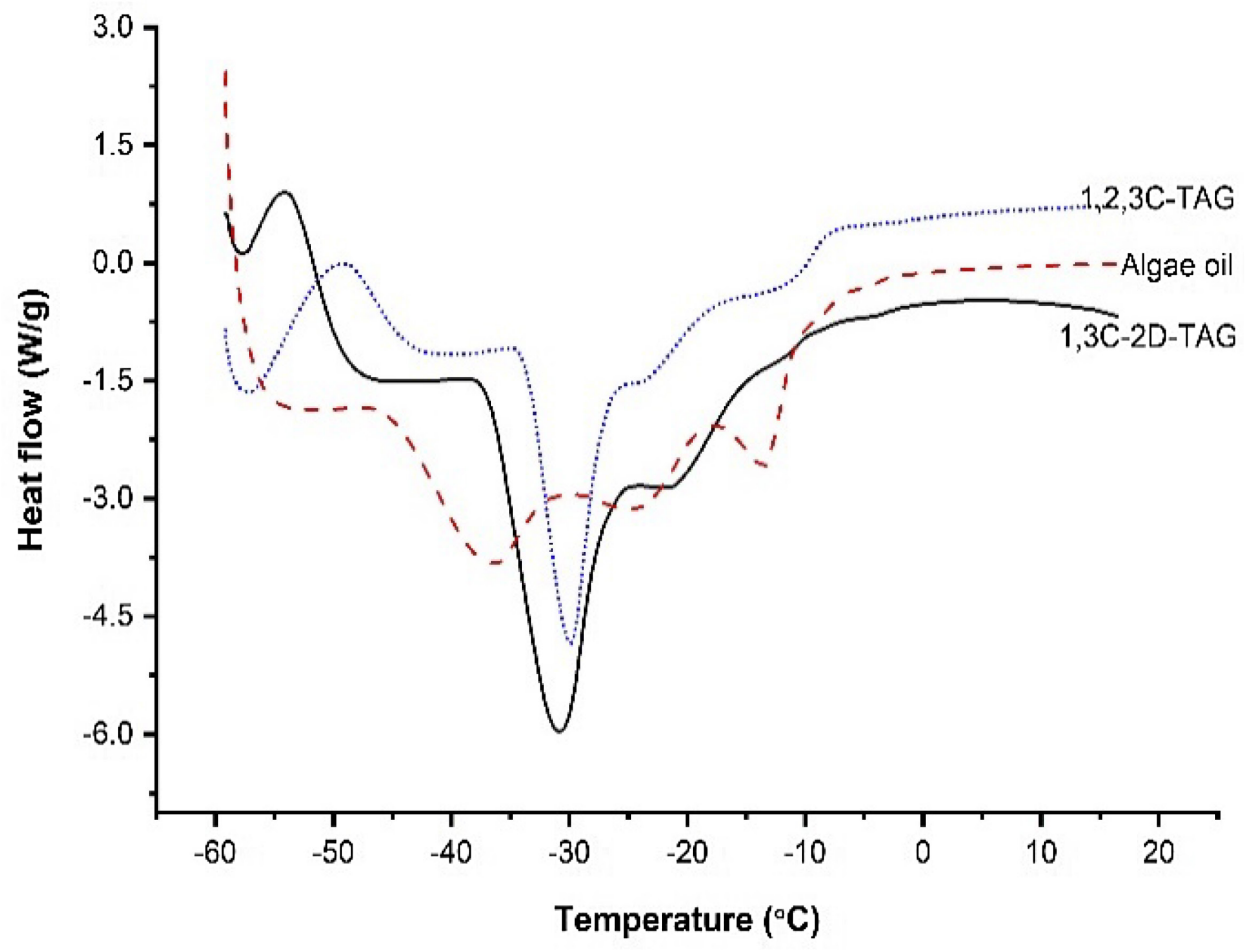
Melting and crystallization temperature profiles of triglyceride caprylate and MLM‐type structured lipids before and after modification.

The crystalline morphology of oils is closely related to their chemical structure and composition. Zou et al. (2016) reported that only oils with melting points less than their physiological temperature (36.6°C–37.3°C) can be quickly emulsified and absorbed by the digestive system. Therefore, the melting and crystallization properties of oils and fats are essential for studying in vivo digestion and absorption. Figure 2 demonstrates that the melting and crystallization temperatures of algal oil, 1,3C‐2D‐TAG, and 1,2,3C‐TAG were lower than their physiological temperature. For algal oil, three crystallization peaks were observed at −36.47°C, −24.22°C, and −12.63°C; the peak at −36.47°C was the largest, indicating that algal oil begin to crystalize at −12.63°C; the crystallization state became evident at −36.47°C. The three continuous crystallization peaks in algal oil observed in the present study may be ascribed to the coagulation and formation of a physical cross‐linking network of the molecular chains in high‐molecular‐weight TAG. The density and strength of condensation mainly depend on the temperature. When the temperature of the triglyceride mixture changes rapidly, the molecular chains tend to move and change to a low‐energy state, which inevitably results in their entanglement to form a new coagulation structure while simultaneously releasing energy (Xie & Hu, 2016).

After modifying the triglycerides with octanoic acid at *sn*‐1,3 position, their melting and crystallization behaviors considerably changed compared with those before modification. 1,3C‐2D‐TAG exhibited a low melting peak at −54.28°C and a high crystallization peak at −31.64°C, suggesting that 1,3C‐2D‐TAG can easily undergo solid–liquid transition. Furthermore, the decrease in its long‐chain PUFA content is conducive to the occurrence of glass transition; it also has a high degree of crystallization at a low temperature. Some studies have confirmed that when the unsaturated fatty acid content and number of carbon atoms are higher, the oil tends to crystallize at a low temperature; this modification can enhance the crystallization properties of the oil (Pang et al., 2013).

The melting peak of 1,2,3C‐TAG was observed at −48.95°C, which had shifted to the right by 6°C compared with that of 1,3C‐2D‐TAG; this indicates that 1,2,3C‐TAG has better plasticity than 1,3C‐2D‐TAG. Although octanoic acid is present at the *sn*‐2 position of triglycerides, the crystallization peaks of both triglycerides were at approximately −30°C. These results suggest that the insertion of fatty acids at the *sn*‐2 position has no effects on the crystallization temperature of triglycerides; however, the insertion of long‐chain unsaturated fatty acids can result in a significant increase in their crystallization degree. The presence of fatty acids (octanoic acid) at the *sn*‐1 and *sn*‐3 positions could remarkably affect the crystallization and melting characteristics of triglycerides, both of which are considered important for their stability and commercialization.

### 3.5 Effects of structured lipids on body weight of mice

**FIGURE 3:**
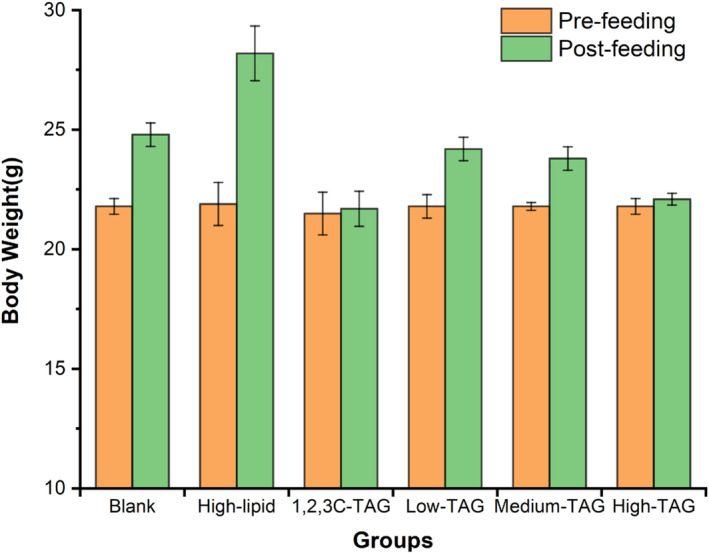
Effects of differently structured lipids on the body weight of C57BL/6 mice. Each column represents the mean of three replicates. Bars indicate standard errors.

Obesity is closely associated with atherosclerosis and hyperlipidemia, and body weight most intuitively reflects the degree of obesity in rats (Álvarez & Akoh, 2016). After feeding for 6 weeks, the weight of C57BL/6 mice increased compared with that before feeding. Furthermore, the weight of mice in the control group after feeding increased by 3.00 g, which was significantly higher than that before feeding (*p* < .05). Compared with that before feeding, the body weight of mice in the high‐lipid group after feeding increased by 6.30 g and was significantly higher than that in the blank group both before and after feeding (*p* < .05). These results indicated that the animal model was successfully developed. The mice fed with 10% 1,2,3C‐TAG exhibited no significant changes in body weight, indicating that the 1,2,3C‐TAG structured lipid can effectively prevent and control obesity in mice. The body weight of mice in the experimental groups fed with food supplemented with low, medium, and high doses of 1,3C‐2D‐TAG increased by 11.32% (*p* < .05), 8.90% (*p* < .05), and 1.33% (*p* < .05), respectively. Mice in the 1,3C‐2D‐TAG medium‐dose and high‐dose groups weighed significantly lower than those in the high‐lipid group, indicating that, to a certain extent, 1,3C‐2D‐TAG could reduce the weight gain of C57BL/6 mice (Figure 3). Studies have also revealed that the addition of fish oil, rapeseed oil, and MLM‐type structured lipids can remarkably influence the growth indices of rainbow trout. Furthermore, the body weight and fat content of rainbow trout fed with structured lipids were found to be significantly lower than those of rainbow trout fed with fish oil and rapeseed oil (Nielsen et al., 2005).

The presence of liver hyperplasia and hypertrophy can be used to determine the extent of liver damage. The liver index (LW/BW) of the blank group was lower than those of the high‐lipid and other experimental groups (Table 4). The liver index of the high‐lipid group was 4.86%, which was significantly higher than those of the 1,3C‐2D‐TAG high‐dose, medium‐dose, and low‐dose experimental groups (*p* < .05). This indicated that 1,3C‐2D‐TAG could inhibit proliferation or hypertrophy of the liver in mice. The kidney index of the high‐lipid group was lower than that of the blank group (*p* < .05), and the high‐lipid group showed a kidney index similar to those of the low‐dose and high‐dose groups (*p* > .05). Thus, the increased weight of mice due to 1,3C‐2D‐TAG was not controlled via kidney metabolism. The kidney index of the 1,2,3C‐TAG group was significantly higher than that of the blank and other experimental groups (*p* < .05). We observed that 1,2,3C‐TAG influences the weight control of mice and inferred that 1,2,3C‐TAG can affect the body weight of mice primarily via kidney metabolism. Nagata et al. (2003) reported that MLM‐type structured lipids containing medium‐chain fatty acids and linoleic acid at the *sn*‐1,3 and *sn*‐2 positions, respectively, are the preferred substrates for promoting energy supply to the pancreas; moreover, the fatty acids at *sn*‐2 position are not involved in promoting energy supply. However, Druschky and Pscheidl (2000) reported that the average body weight and nitrogen balance of mice fed with the *sn*‐2 omega‐3 were lower than those of mice in the control group. Furthermore, the average body weight and nitrogen balance did not significantly decrease after omega‐3 was replaced with omega‐6 at the same position. Therefore, structured fats containing *sn*‐1,3 fatty acids significantly affect lipid metabolism in animals, and unsaturated *sn*‐2 fatty acids exert certain effects on body weight.

**TABLE 4:** Effects of differently structured lipids on body weight in C57BL/6 mice.

Group	Liver weight/body weight (%)	Kidney weight/body weight (%)
Blank	3.92 ± 0.13^c^	1.04 ± 0.05^b^
High‐lipid	4.86 ± 0.18^a^	0.95 ± 0.03^c^
1,2,3C‐TAG	4.57 ± 0.26^ab^	1.40 ± 0.05^a^
Low‐dose TAG	4.24 ± 0.09^bc^	0.90 ± 0.06^c^
Medium‐dose TAG	4.26 ± 0.12^bc^	1.02 ± 0.04^b^
High‐dose TAG	4.32 ± 0.11^b^	1.00 ± 0.04^bc^

*Note*: Values are means ± standard deviation. Mean values in the same row with different superscript letters are significantly different (*p* < .05).

### 3.6 Effects of structured lipids on lipid metabolism in mice

**FIGURE 4:**
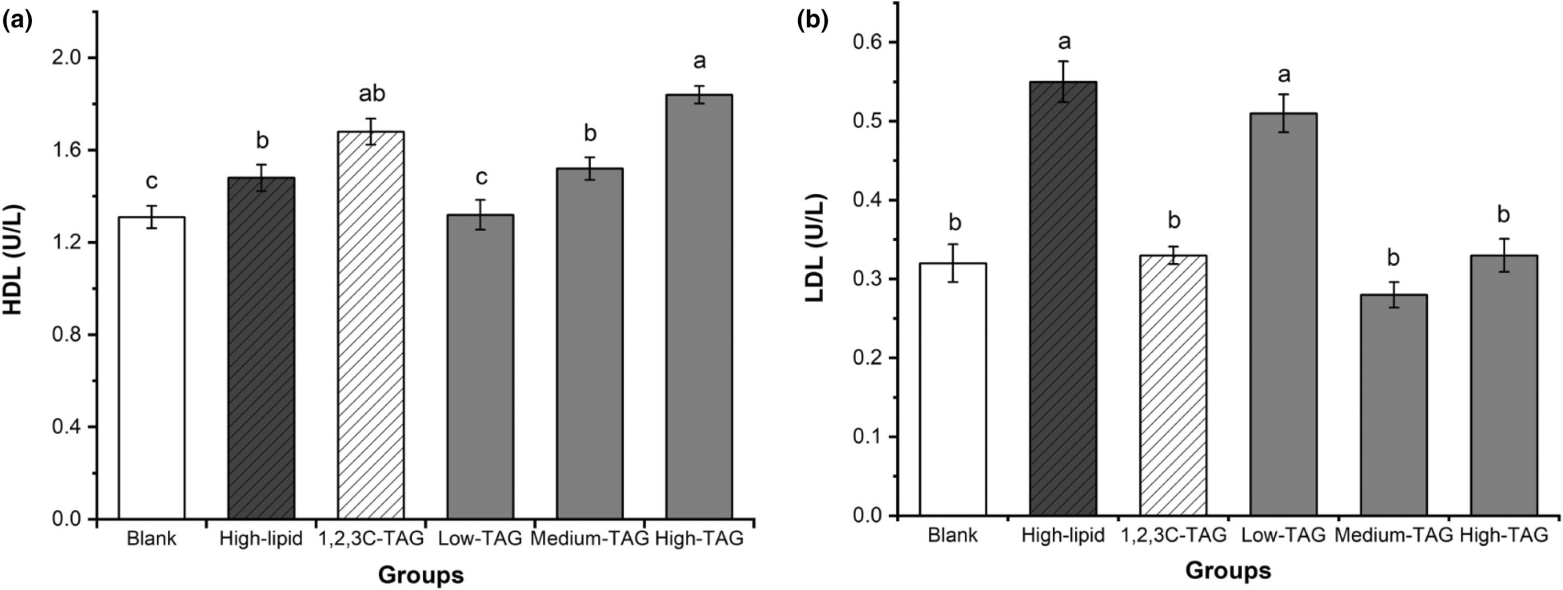
Effects of differently structured lipids on the levels of high‐density lipoprotein (HDL‐C) and low‐density lipoprotein cholesterol (LDL‐C) in C57BL/6 mice. Each column represents the mean of three replicates. Bars indicate standard errors. Values followed by different letters are significantly different (*p* < .05).

HDL is a complex lipoprotein comprising lipids, proteins, and their regulatory factors. It primarily exhibits antiatherosclerosis properties and protects against coronary heart disease. As observed in Figure 4, after 6 weeks of feeding, the HDL‐C levels in the blank group were lower than those in the high‐lipid, 1,2,3C‐TAG, and 1,3C‐2D‐TAG groups; HDL‐C levels in the high‐dose group were significantly higher than those in the 1,2,3C‐TAG, medium‐dose, and low‐dose groups (*p* < .05). These results indicated that a high dose of 1,3C‐2D‐TAG can increase HDL‐C levels in C57BL/6 mice in a dose‐dependent manner. The 1,2,3C‐TAG group also exhibited significantly higher HDL‐C levels than the blank group (*p* < .05). Moreira et al. (2017) studied the mice fed with MLM‐type structured lipids and revealed significantly decreased levels of adipose tissue, total cholesterol, LDL cholesterol, and liver weight, whereas an increased amount of fat was noted in the feces along with significantly increased level of HDL cholesterol in plasma.

In contrast to HDL‐C, the levels of LDL‐C in the high‐lipid and low‐dose 1,3C‐2D‐TAG groups were significantly higher than those in other groups (*p* < .05). LDL‐C levels in the high‐dose 1,3C‐2D‐TAG and 1,2,3C‐TAG groups did not differ significantly. Furthermore, the LDL‐C levels in the medium‐dose 1,3C‐2D‐TAG group were the lowest, indicating that medium and high doses of 1,3C‐2D‐TAG could control the levels of LDL‐C in mice. Studies have revealed that weight gain is low after the mice are fed with C22:0 structural fats. Although the signs of toxicity or diarrhea were not observed, the amount of lipid excreted in animal feces and the levels of HDL increased significantly. The study concluded that structured lipids played a crucial role in preventing or treating obesity (Moreira et al., 2017). Altogether, 1,3C‐2D‐TAG plays a role in improving atherosclerosis induced by a high‐lipid diet in mice, especially in mice in the medium‐dose group.

### 3.7 Effects of structured lipids on total cholesterol and triglycerides in mice

**FIGURE 5:**
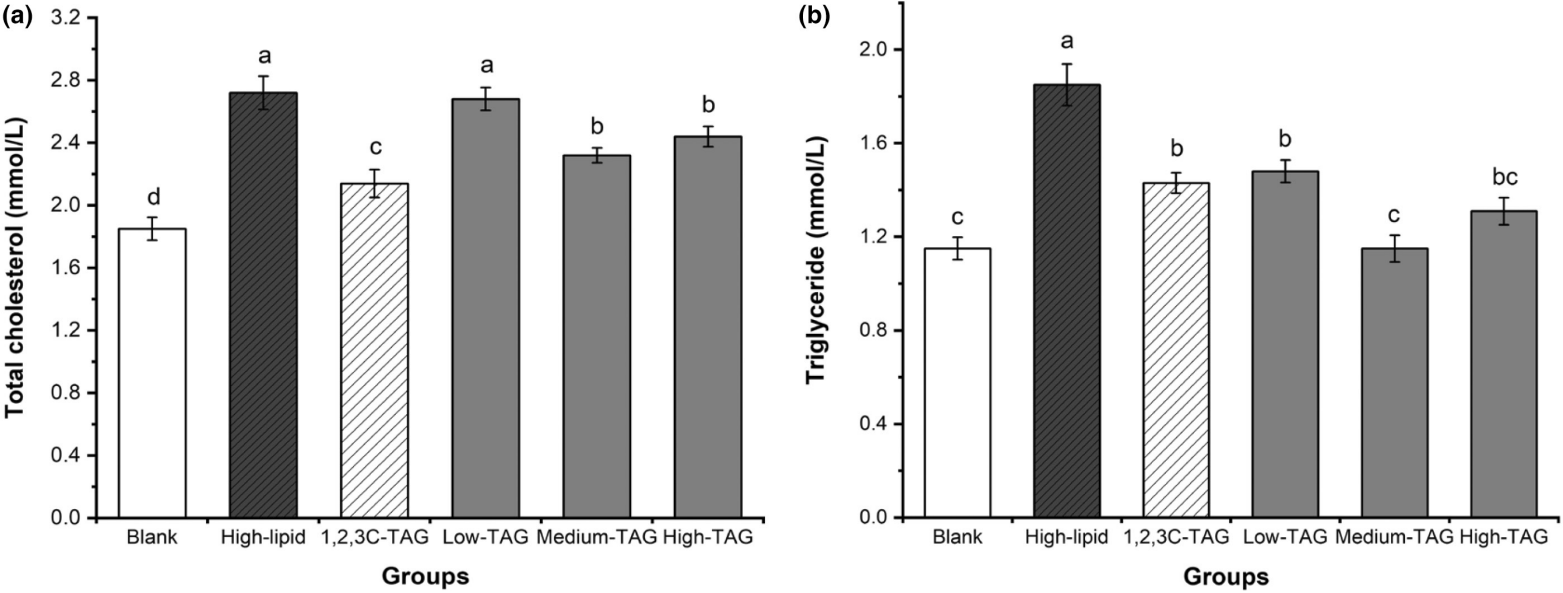
Effects of differently structured lipids on total cholesterol and triglycerides in C57BL/6 mice. Each column represents the mean of three replicates. Bars indicate standard errors. Values followed by different letters are significantly different (*p* < .05).

Total cholesterol and triglycerides in the high‐lipid group were significantly higher than those in the blank group, indicating that a high‐lipid diet could effectively lead to an increase in the levels of total cholesterol and triglycerides in blood of the mice. The low‐dose 1,3C‐2D‐TAG group exhibited lower total cholesterol and triglyceride levels than the high‐lipid group, but the difference was not obvious (*p* > .05). This result indicates that 1,3C‐2D‐TAG could inhibit the increase in the levels of total cholesterol and triglycerides in the mice (Figure 5). Nagata et al. (2004) studied the effects of MLM‐type structured lipids on serum and liver lipids in rats. They revealed that the serum cholesterol and lipid concentrations in rats fed with MLM‐type structured lipids were significantly lower than those of rats in the control group that were fed soybean oil. Notably, the effect of 1,2,3C‐TAG on total cholesterol and triglycerides in mice is similar to that of 1,3C‐2D‐TAG. 1,2,3C‐TAG inhibits weight gain in mice, and some studies have also revealed that diets rich in *sn*‐1 and *sn*‐3 diacylglycerol can significantly reduce the levels of total cholesterol, triglycerides, and glucose in animal plasma (Eom et al., 2010). Medium‐chain fatty acids obtained via enzymatic hydrolysis at 3‐position can rapidly supply energy to the body and are not mixed with chyle particles. Most medium‐chain fatty acids cannot be stored in adipose tissues, thus reducing the probability and risk of hyperlipidemia in mice (Kahveci et al., 2015). Because the effects of 1,2,3C‐TAG and high‐dose 1,3C‐2D‐TAG on total cholesterol and triglycerides in the mice were similar, we hypothesized that the medium‐chain fatty acids at *sn*‐1,3 position were the primary reason leading to obesity, and the fatty acids at *sn*‐2 position did not affect the regulation of obesity.

### 3.8 Effect of structured lipids on BUN in mice

**FIGURE 6:**
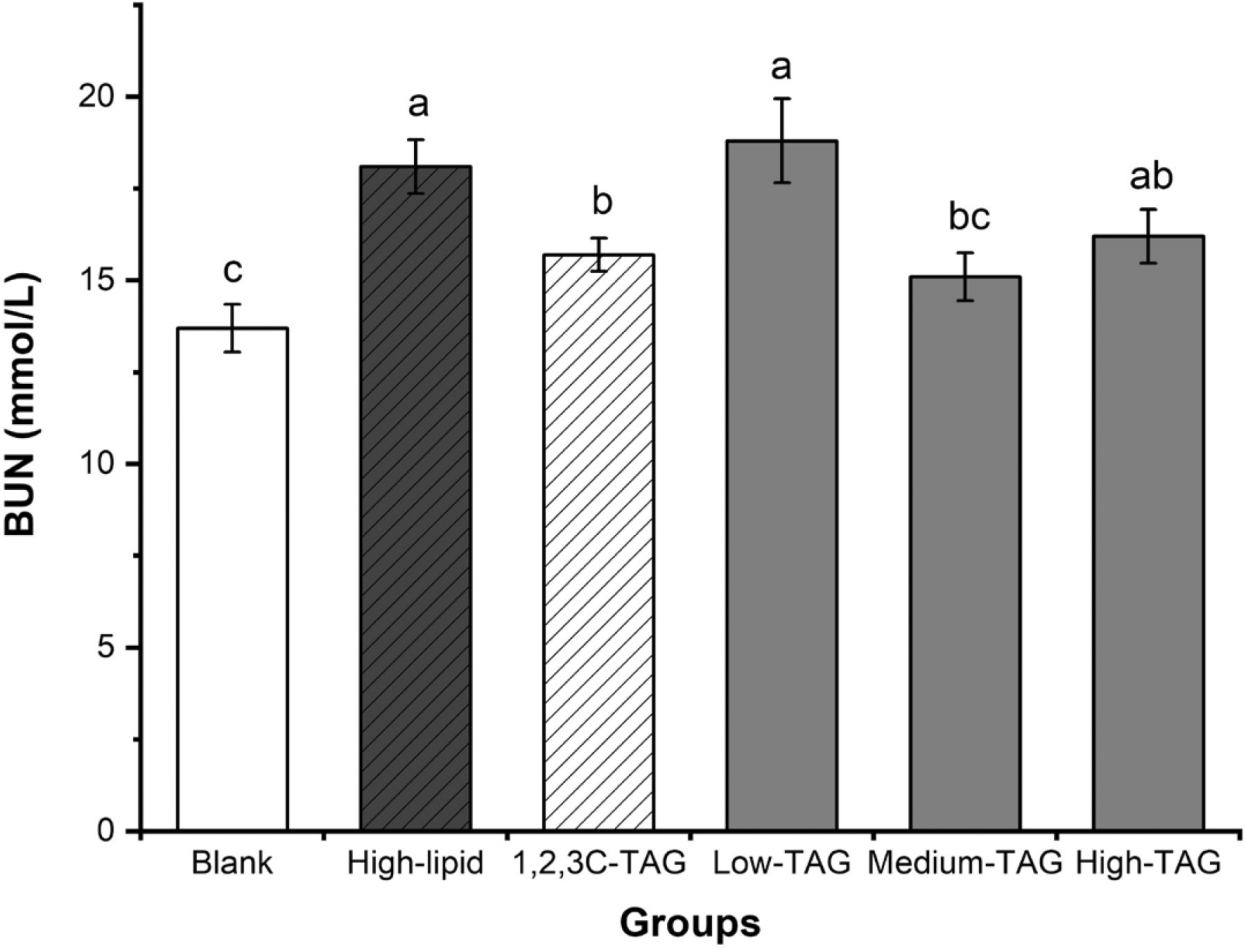
Effects of differently structured lipids on blood urea nitrogen (BUN) in C57BL/6 mice. Each column represents the mean of three replicates. Bars indicate standard errors. Values followed by different letters are significantly different (*p* < .05).

BUN is the primary end product of protein metabolism that is excreted from the body via glomerular filtration. It is a reference index that can be used to indicate kidney function damage. The concentration of BUN in the high‐lipid group (16.40 mmol/L) was higher than that in the blank group (13.82 mmol/L), indicating that a high‐lipid diet affects kidney function (Figure 6). Compared with that in the high‐lipid group, the level of BUN in the low‐dose 1,3C‐2D‐TAG group did not change significantly. BUN levels (15.13 mmol/L) in the medium‐dose 1,3C‐2D‐TAG group were significantly lower than those in the high‐lipid group (*p* < .05), indicating that a medium dose of 1,3C‐2D‐TAG cannot cause nephrotoxicity in mice.

**FIGURE 7:**
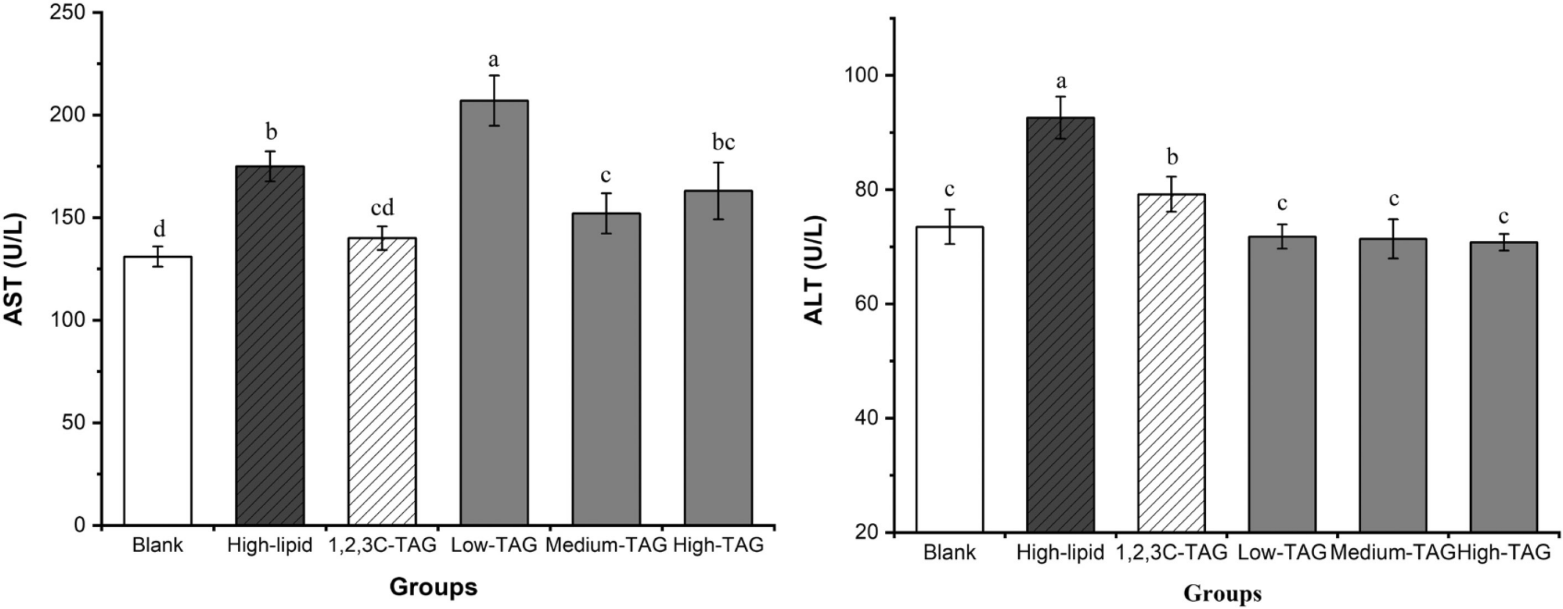
Effects of differently structured lipids on aspartate aminotransferase (AST) and alanine aminotransferase (ALT) in C57BL/6 mice. Each column represents the mean of three replicates. Bars indicate standard errors. Values followed by different letters are significantly different (*p* < .05).

The liver is the primary organ for lipid, carbohydrate, and protein metabolism. AST and ALT levels are representative indicators of liver function because they directly reflect the degree of damage to liver cells; the higher the levels of these compounds, the greater the degree of damage (Hu et al., 2011). Figure 7 indicates that AST and ALT levels in the high‐lipid group were significantly higher than those in the control group (*p* < .05), indicating that high‐lipid diet‐induced hyperlipidemia can cause damage to the liver and affect lipid metabolism in mice. Compared with those in the blank group, AST levels in the 1,2,3C‐TAG group were significantly lower than those in the low‐dose 1,3C‐2D‐TAG group, but they were not significantly different (*p* > .05) from those in the medium‐ and high‐dose groups. Similarly, ALT levels in the medium‐ and high‐dose groups were not significantly different (*p* > .05). These results indicated that medium and high doses of 1,3C‐2D‐TAG cannot cause liver toxicity in mice. Sengupta and Ghosh (2010) also reported that a certain dose of MLM‐type structured lipids can partially improve the characteristics of blood components in rats by reducing the content of transaminase.

### 3.9 Effects of structured lipids on livers and kidneys of mice

**FIGURE 8:**
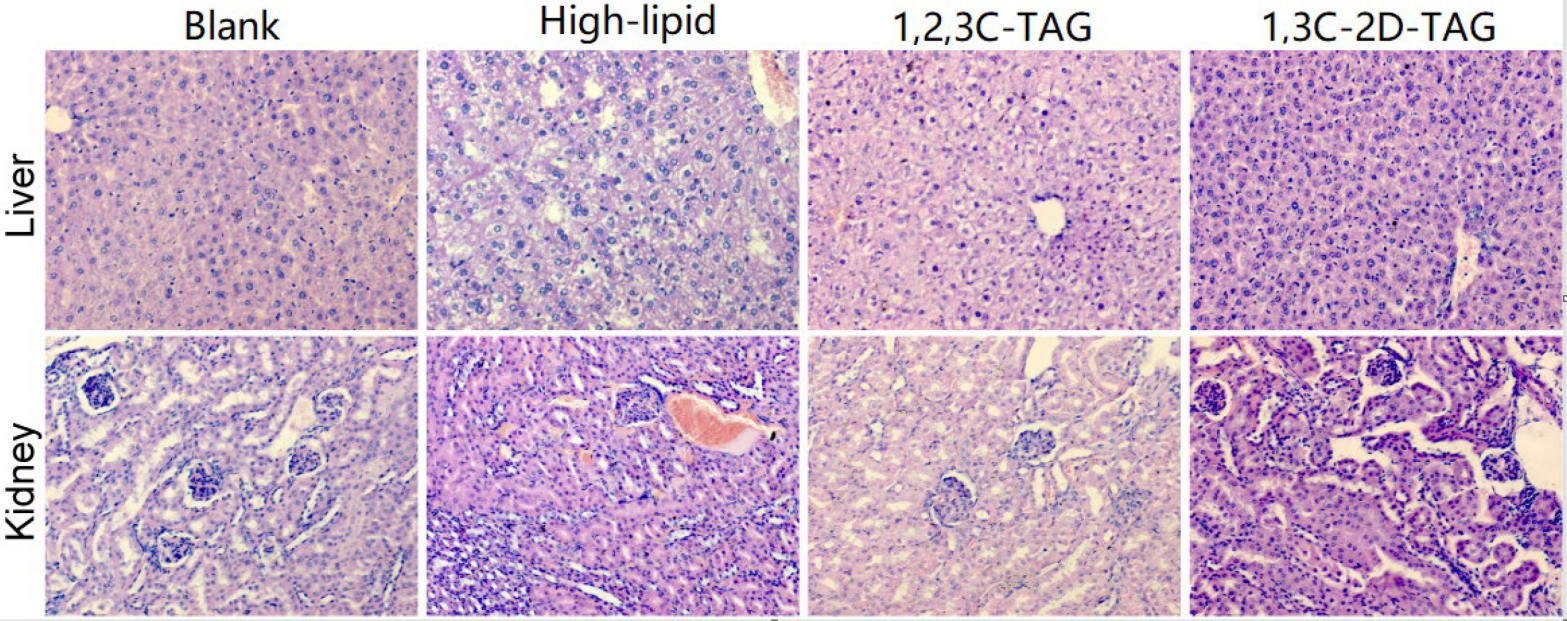
Effects of differently structured lipids on the morphologies of liver and kidney tissues of C57BL/6 mice.

We further studied the effects of 1,3C‐2D‐TAG on the liver and kidney tissues of the mice. As shown in Figure 8, in the pathological section of livers in the blank group, individual hepatocytes were observed to be complete; the boundaries were clear; the cells were intact with undamaged nuclei, exhibiting similar sizes and round shapes, and they were evenly arranged around the central vein. In the high‐lipid group, the nuclei were severely damaged and arranged in a disordered manner, and the tissue structures were loose with a large area of vacuoles. The liver cells of the 1,3C‐2D‐TAG and 1,2,3C‐TAG groups were slightly enlarged but arranged relatively evenly, and the tissue structures were dense with few or no vacuoles. The solubilities of medium‐chain fatty acids are higher than those of long‐chain fatty acids; thus, they can directly provide energy to the liver. However, long‐chain fatty acids are primarily absorbed and metabolized by the lymphatic system after being transported to intestinal wall micelles (Sengupta & Ghosh, 2011). Therefore, the metabolism and absorption of *sn*‐1,3 medium‐chain fatty acids are primarily concentrated in the liver, and the medium‐chain fatty acids can accelerate energy metabolism. In the high‐lipid group, the boundaries of liver cells were clear and those of the kidney cells were slightly enlarged; furthermore, the nuclei were small in size with a thick membrane. In addition, the chromatin became denser, the cytoplasm was filled with a large number of different‐sized fat droplets, the nucleus was squeezed to one side, and several fat droplets were observed within the field of vision. In the 1,3C‐2D‐TAG group, some kidney cells were damaged; however, the degree of injury was relatively low, and some cells appeared to exhibit slight edema.

**FIGURE 9:**
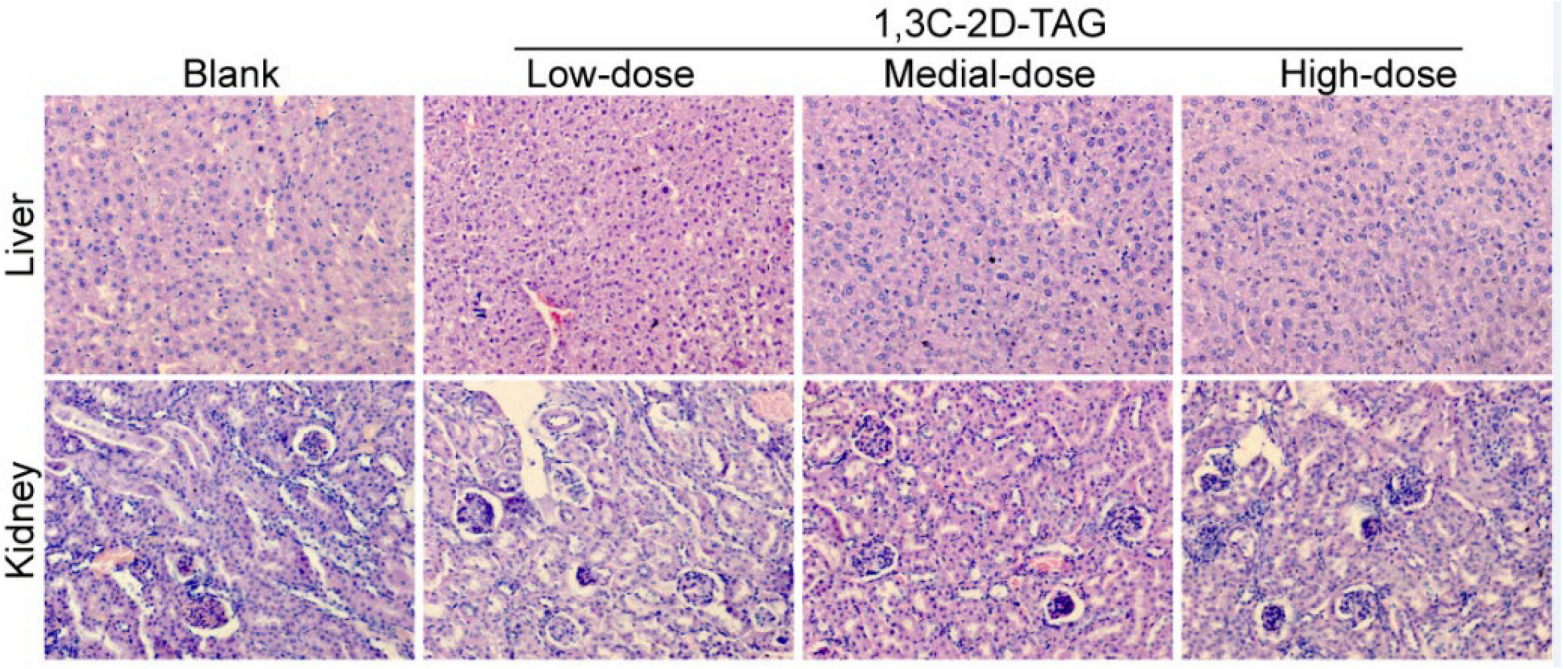
Effects of 1,3C‐2D‐TAG at different doses on the morphologies of liver and kidney tissues of C57BL/6 mice.

As shown in Figure 9, hepatocytes in the liver and kidney sections in the low‐dose group were arranged in a more disordered manner compared with those in the high‐lipid group; in addition, the nuclei were of different sizes, cell boundaries were more obvious, and there were only a few vacuoles in the low‐dose group. In the medium‐dose group, the hepatocytes were orderly and closely arranged, and the intercellular space was obvious. The cells surrounding the central vein were orderly arranged, and other cells were distributed in a regular and sporadic manner. The morphologies of hepatocytes in the high‐dose group were similar to those in the blank group, with a relatively uniform arrangement. The fat droplets were relatively small, and their numbers were also relatively low. The boundaries of hepatocytes were clear and the hepatic sinuses were visible. The sizes and numbers of fat droplets in hepatocytes in the high‐dose 1,3C‐2D‐TAG group did not significantly differ from those in the blank group, indicating that the accumulation of triglycerides in hepatocytes was reduced after triglyceride synthesis was controlled. In contrast to those in the low‐dose group, the nuclear membranes of kidney cells in the high‐dose 1,3C‐2D‐TAG group were clear, smooth, and intact. The nucleoli were clear and visible, cytoplasmic staining was fresh and uniform, and the central vein and venous sinus space did not exhibit any dilation or congestion. Fibrous tissue proliferation and inflammatory cell infiltration were not observed, similar to the morphologies of kidney cells in the blank group. Thus, the effects of 1,3C‐2D‐TAG on the liver and kidney cells were also dose dependent.

### 3.10 Effect of structured lipids on leptin in mice

**FIGURE 10:**
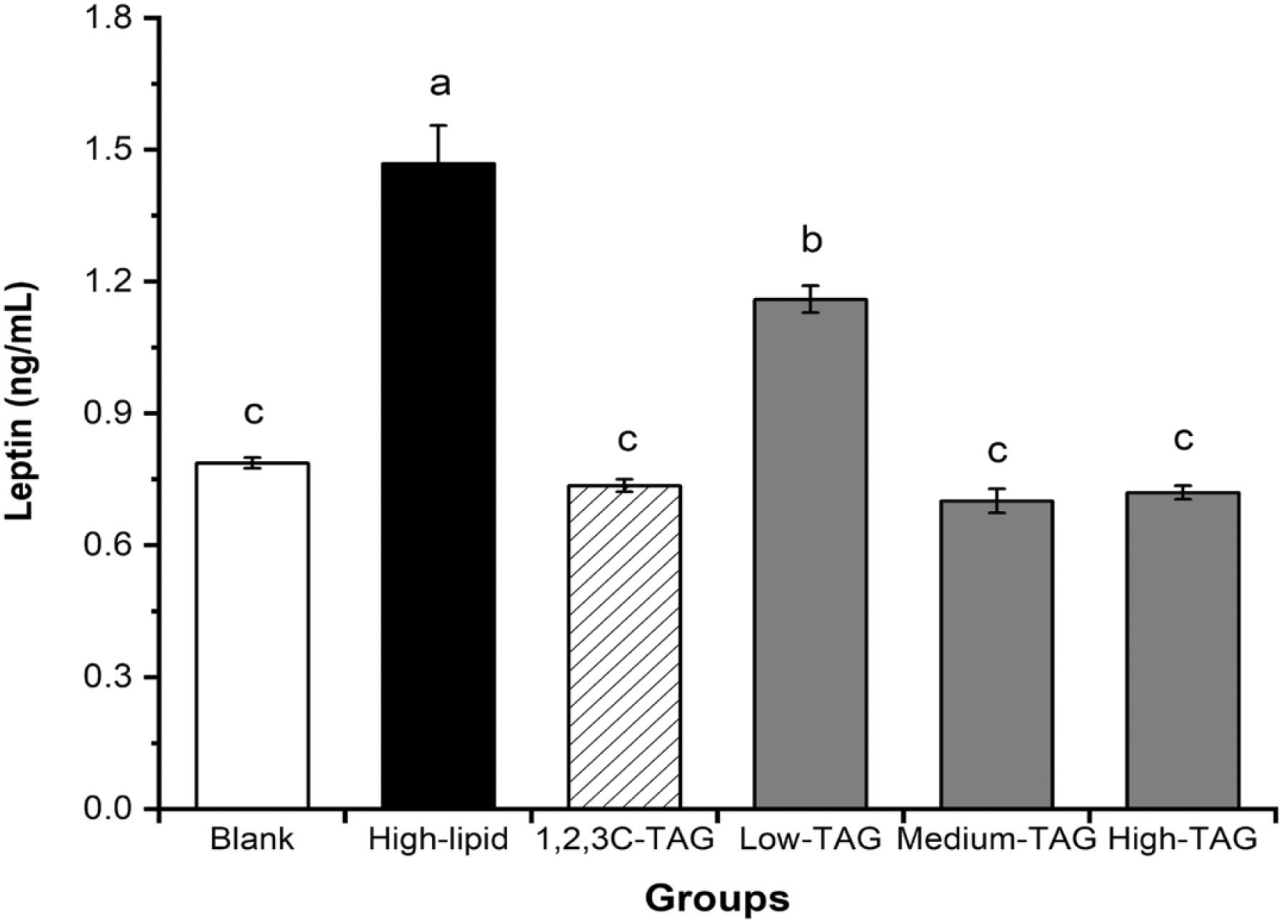
Effects of differently structured lipids on leptin in C57BL/6 mice. Each column represents the mean of three replicates. Bars indicate standard errors. Values followed by different letters are significantly different (*p* < .05).

Leptin is a hormone secreted by adipose tissues. It binds to the leptin receptor on hypothalamic nerve cells, resulting in a series of physiological effects, including satiety responses, which limit the body's energy intake and consumption that leads to a balance in body weight and fat. As shown in Figure 10, the level of leptin in the high‐lipid model group was 1.47 ng/mL, which is significantly higher than that in the blank and experimental groups (*p* < .05). Thus, obesity can result in hormonal imbalance to a varying degree, and mice are unable to make appropriate choices in terms of energy intake. However, the leptin levels in the high‐dose group were lower than those in the low‐dose group. Leptin regulates the appetite, thereby regulating body weight and fat homeostasis. Studies have reported the presence of substances that are antagonistic to leptin function (triglycerides) in the blood of patients with obesity. Studies have also revealed that leptin mediates abnormal neural signal transduction pathways (the transport efficiency of leptin receptor is decreased), causing abnormalities in the body's self‐regulation system to varying degrees. Therefore, the level of leptin in patients with obesity is often very high, resulting in the onset of leptin resistance. The differences between the levels of leptin in the 1,2,3C‐TAG group and those in blank, medium‐dose 1,3C‐2D‐TAG, and high‐dose 1,3C‐2D‐TAG groups were not significant, indicating that medium‐chain fatty acids can alleviate leptin resistance, thus playing a positive role in regulating and controlling body fat homeostasis. Therefore, medium and high doses of 1,3C‐2D‐TAG may significantly reduce the diet‐induced leptin concentration in rats, effectively alleviating obesity‐induced leptin resistance.

The types and positions of fatty acids in triglycerides are important factors for controlling efficient fatty acid utilization. In this process, MLM‐type structured lipids containing *sn*‐2 long‐chain PUFAs and *sn*‐1,3 fatty acids in the digestive tract are preferentially hydrolyzed by pancreatic lipase to produce free fatty acids and 2‐MAG, which can be effectively absorbed. The 1,3C‐2D‐TAG‐type structured lipids containing *sn*‐1,3 medium‐chain fatty acids and other fatty acid types are present in low volumes but exhibit high solubility. Because of its high hydrophilicity, fat cannot be easily converted into TAG and is primarily transported to the liver through portal vein (nonlymphatic system); however, it has been found to be more easily digested and absorbed for quickly supplying energy in vivo (without forming saponification complexes with calcium or magnesium ions). Medium‐chain fatty acids cannot be stored in adipose tissues; thus, they can reduce the risk of hyperlipidemia in mice. Hydrolyzed 2D‐MAG with long‐chain fatty acids cannot be oxidized under the presence of glycerol, but they can be absorbed by mucosal cells in the small intestine and can easily pass through the intestinal wall. Therefore, the grafting of long‐chain unsaturated fatty acids at *sn*‐2 position of TAG not only facilitates the effective utilization of the energy supplied by medium‐chain fatty acids in a timely manner but also helps overcome the difficulty in absorbing long‐chain PUFAs (Figure 10).

## 4 CONCLUSIONS

We investigated the effects of different types and doses of structured lipids on body weight and levels of fat, total cholesterol, triglycerides, BUN, leptin, AST, and ALT in C57BL/6 mice using an obese animal model. The data revealed that the mice in the low‐dose, medium‐dose, and high‐dose 1,3C‐2D‐TAG groups exhibited increased weight. The body weight of mice in the medium‐dose and high‐dose 1,3C‐2D‐TAG groups was significantly lower than that in the high‐lipid model group, except for that of mice in the low‐dose 1,3C‐2D‐TAG group. The results also revealed that 1,3C‐2D‐TAG could reduce the weight gain of C57BL/6 mice, whereas it could inhibit hyperplasia or hypertrophy in the liver of the mice to a certain extent. Furthermore, 1,3C‐2D‐TAG could ameliorate atherosclerosis in the high‐lipid group fed with a high‐lipid diet and balanced lipid metabolism by reducing LDL‐C levels and increasing HDL‐C levels. The effects were especially prominent in mice in the medium‐dose group because they exhibited superior lipid regulation. Medium and high doses of 1,3C‐2D‐TAG structured lipids could significantly reduce diet‐induced leptin concentrations in the mice and could effectively alleviate obesity‐induced leptin resistance. Taken together, grafting long‐chain unsaturated fatty acids onto *sn*‐2 position of TAG can not only ensure the effective and timely supply of energy by medium‐chain fatty acids but also helps in overcoming the difficulty in absorption of long‐chain PUFAs.

## CONFLICT OF INTEREST STATEMENT

The authors declare no conflict of interest or relationship.

## INSTITUTIONAL REVIEW BOARD STATEMENT

The study was conducted according to the guidelines of the Declaration of China, and approved by the Beijing Technology and Business University.

## Data Availability

The datasets generated or analyzed during this study are available from the corresponding author on reasonable request.
